# Functional and Phylogenetic Structure of Forest Bird Assemblages Along an Afrotropical Elevational Gradient

**DOI:** 10.1002/ece3.72065

**Published:** 2025-08-27

**Authors:** Riccardo Pernice, Ondřej Sedláček, Tomáš Albrecht, Oldřich Tomášek, Ondřej Kauzál, Tereza Kauzálová, Francis Njie Motombi, Francis Luma Ewome, Michal Ferenc, Kryštof Chmel, Jiří Mlíkovský, Jan Riegert, Solange Mekuate Kamga, David Hořák

**Affiliations:** ^1^ Department of Ecology, Faculty of Science Charles University of Prague Prague Czech Republic; ^2^ Czech Academy of Sciences, Institute of Vertebrate Biology Brno Czech Republic; ^3^ Department of Zoology, Faculty of Science Charles University of Prague Prague Czech Republic; ^4^ Mt Cameroon National Park Buea Cameroon; ^5^ Faculty of Science University of South Bohemia Ceske Budejovice Czech Republic; ^6^ Faculty of Science, Laboratory of Applied Biology & Ecology University Dschang Dschang Cameroon

**Keywords:** Africa, community assembly, competition, elevational gradient, environmental filtering, functional diversity, guild assembly rule, phylogenetic diversity, tropical forest

## Abstract

Elevational gradients offer valuable opportunities to investigate biodiversity patterns and the ecological and evolutionary processes that shape them. Although tropical mountains are recognized as biodiversity hotspots, the various dimensions of biodiversity in these systems, particularly in equatorial Africa, remain poorly understood. In this study, we examined the functional (FD) and phylogenetic diversity (PD) of bird assemblages along a primary forest elevational gradient in Cameroon, West‐Central Africa, spanning from lowland forests to the treeline (~2300 m a.s.l.). We analyzed how FD and PD vary with elevation and tested the roles of abiotic filtering and biotic interactions, such as competition, in community assembly. Additionally, we assessed whether taxonomic diversity (TD) increases through niche packing or expansion, based on morphological and resource‐use traits. Using null models and bird occurrence data, we inferred the drivers of FD and PD patterns and evaluated whether species in more diverse assemblages occupied novel functional space compared to less diverse assemblages. Our results showed that both functional richness and TD declined with elevation, whereas functional nearest neighbor distance, functional evenness, and mean nearest taxon distance increased. Traits related to resource use suggested that bird species at higher elevations were functionally less similar than expected by chance, partially supporting the influence of competition consistent with the guild assembly rule. Phylogenetic clustering observed at both low and high elevations pointed to independent species radiations, likely shaped by historical forest dynamics. In species‐rich lowland assemblages, we found evidence of niche packing, suggesting increased specialization or niche overlap. In contrast, niche expansion appeared to contribute to higher TD at elevated sites. Overall, our findings indicate that while abiotic filters along forested elevational gradients and competition in lowland forests play roles in shaping avian diversity, they are not the sole or dominant mechanisms. Nonetheless, partial support for competition aligns with theoretical expectations under the guild assembly framework.

## Graphical abstract

On Mt. Cameroon, functional richness and taxonomic diversity decreased with elevation, while functional nearest neighbor distance, functional evenness, and mean nearest taxon distance showed positive trends. Traits related to resource use indicated that higher‐elevation species were functionally less similar than expected by chance, supporting partially competition under the guild assembly rule. Phylogenetic clustering at low and high elevations suggested independent species radiation linked to historic forest dynamics. Low‐elevation, species‐rich assemblages displayed niche packing, while niche expansion contributed to TD increases at higher elevations.

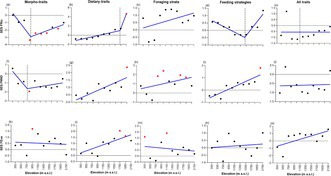

## 1 Introduction

Understanding the factors that influence community assembly and species' distribution along elevational gradients poses a significant challenge to ecology, biogeography, and conservation biology (Graham et al. 2014; Hubbell 2011; Quintero and Jetz 2018; Si et al. 2017). Recently, there has been a growing emphasis on exploring multiple dimensions of diversity to understand the ecological and evolutionary processes shaping the observed biodiversity patterns (Aros‐Mualin et al. 2021; Ding et al. 2019; Lamanna et al. 2014). Unlike taxonomic diversity (TD), phylogenetic diversity (PD) and functional diversity (FD) focus on species' attributes within an assemblage and quantify the degree of similarity among species (Albert et al. 2012; Li et al. 2019; Pavoine and Bonsall 2011). While PD provides means to measure and understand the differences in evolutionary history among species (Cadotte et al. 2013; Webb et al. 2002), FD focuses on measuring species' ecological differences and describing their distribution within a niche space based on functional traits (Dehling and Stouffer 2018; Petchey and Gaston 2006). Thus, comparing functional and phylogenetic dimensions of biodiversity across spatial gradients can help uncover the distinct processes driving the origin and maintenance of biodiversity (Stegen and Hurlbert 2011).

Within the conventional framework of community assembly, species coexistence in a given community is influenced by niche‐based deterministic processes (i.e., interspecific competition and environmental filtering; Cavender‐Bares et al. 2009; Kraft et al. 2015; Webb et al. 2002) or stochasticity (Chase and Myers 2011; Vellend et al. 2014). By investigating patterns of FD and PD and comparing them to null expectations (stochasticity), one can obtain valuable insights into the relative importance of assembly processes shaping community structure along environmental gradients (Mayfield et al. 2005; Spasojevic and Suding 2012; Webb et al. 2002). For instance, when assemblages exhibit a higher degree of functional similarity than null expectations (i.e., functionally clustered assemblages), this is often attributed to environmental filtering (Baraloto et al. 2012; Cavender‐Bares et al. 2009; but see Mayfield and Levine 2010). Environmental filtering is rooted in the stress‐dominance hypothesis (Coyle et al. 2014; Swenson and Enquist 2007; Weiher et al. 1998). According to this hypothesis, the strength of environmental filtering is expected to be highest in conditions characterized by greater levels of abiotic stress. On the other hand, interspecific competition is thought to limit similarity, resulting in resource‐related trait overdispersion (MacArthur and Levins 1967; Abrams 1983; Gotelli and Graves 1996). Assuming niches exhibit phylogenetic conservatism, meaning that closely related species tend to be more ecologically similar than distantly related species, phylogenetic distances among species may reflect evolved ecological differences (Cavender‐Bares et al. 2009; Webb et al. 2002; but see Cadotte et al. 2019; Mason and Pavoine 2013). Consequently, competitive exclusion may lead to assemblages that are phylogenetically overdispersed, as closely related species are more likely to compete and exclude each other (Cavender‐Bares et al. 2009; Violle et al. 2011). In contrast, phylogenetic clustering can indicate community assembly driven by environmental filtering, where closely related species with similar ecological traits are favored by similar environmental conditions (Mayfield and Levine 2010).

Understanding the relationship between FD and TD offers valuable insights into the mechanisms underlying community assembly. Changes in TD may be linked to either trait dispersion (niche expansion) or a denser trait packing within the niche space (MacArthur 1965; Pigot et al. 2016). These two processes are different, but not mutually exclusive. The niche expansion model predicts that increases in TD are associated with the exploitation of novel regions of the niche space (e.g., novel habitats or resources), whereas the niche packing model suggests that higher TD is linked to a dense overlapping of the species' niche or finer specializations (Pellissier et al. 2018; Pigot et al. 2016). Moreover, niche segregation along a species richness gradient may follow Fox's guild assembly rule (Fox 1987). Although conceived for small mammals (Fox 1987; Kohli et al. 2021), the Fox's assembly rule posits that with increasing TD, each functional guild is added until all of them are represented, and then, new species are sequentially added within each guild. This results in the maintenance of maximal niche segregation until a certain threshold, beyond which species are packed within the occupied functional space in proportion to the amount of available resources (Brown et al. 2000; MacArthur 1984; Stevens et al. 2012). Surprisingly, limited progress has been made in understanding how niche expansion, niche packing, and Fox's guild assembly rule shape the structure of assemblages across environmental gradients.

Elevational gradients represent natural laboratories for investigating mechanisms underlying spatial patterns of biodiversity (Guo et al. 2013; Herzog et al. 2005; Vetaas et al. 2019). Elevational gradients offer a unique advantage due to the continuous and rapid variation in environmental conditions over relatively short spatial scales, making mountain ecosystems ideal natural laboratories for testing community assembly theories (Bricca et al. 2019; Cardillo et al. 2008; Cody et al. 1975; Xu et al. 2017). Most of the available knowledge of community structure and evolutionary dynamics comes from temperate systems (Ao et al. 2022; Ding et al. 2021; Lin et al. 2024), and there is only a limited comprehension of the processes governing community assembly in tropical ecosystems, where the number of species is higher and their interactions more complex (Gálvez‐Reyes et al. 2021; Rahbek et al. 2019). This gap extends to tropical Afromontane systems (Maley 1996; Parmentier et al. 2007), especially in the western regions of the continent, as the majority of such studies stem from East Africa (Byamungu et al. 2021; Hořák et al. 2023; Molina‐Venegas et al. 2020; Onditi et al. 2022). To bridge this gap, we used birds as a model system given their well‐documented ecology and phylogeny (Barnagaud et al. 2014; Jetz et al. 2012; Tobias et al. 2022).

**FIGURE 1:**
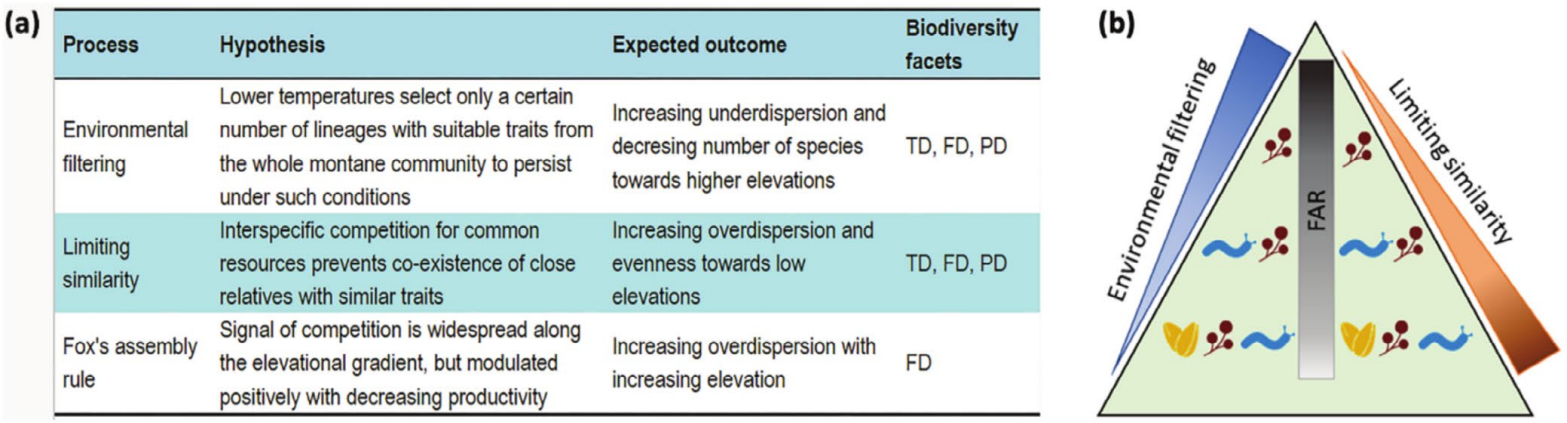
Community assembly hypotheses, expected outcomes, and affected biodiversity facets (FD, functional diversity; PD, phylogenetic diversity; TD, taxonomic diversity) for environmental filtering, limiting similarity, and Fox's assembly rule (FAR) along a tropical elevational gradient (a). Intensity of environmental filtering and limiting similarity over elevation is illustrated by arrow gradients–lighter pointed ends indicate weaker effects, while darker, broader bases represent stronger intensity (b). Here, a rectangular shape with a color gradient represents the pervasive interspecific competition predicted by FAR in response to changes in resource availability.

In this study, we assessed the contribution of environmental filtering and interspecific competition in shaping the structure of avian assemblages with respect to tropical elevation. Specifically, through the lens of FD and PD, we explored a set of mechanistic hypotheses for community assembly (Figure 1a) across ten bird assemblages along a forest elevational gradient (350–2300 m a.s.l.) of Mt. Cameroon (west central Africa). Along tropical elevational gradients such as on Mt. Cameroon, the peak in abiotic stress is expected to occur at high elevations, where environmental filtering may select for adaptations to lower temperatures (Barrio et al. 2013; Coyle et al. 2014; Londoño et al. 2017). By consequence, we expect trait clustering towards higher elevations. Assuming niche conservatism, we predict that a similar pattern would emerge concerning phylogenetic diversity as well (Cavender‐Bares et al. 2009). In contrast, hot and humid conditions at low elevations are expected to favor limiting similarity (sensu MacArthur and Levins 1967) and high interspecific competition, leading to trait and phylogenetic overdispersion (Boyce et al. 2019; Graham et al. 2009; Montaño‐Centellas et al. 2020).

When environmental filtering is weak and competition becomes the dominant force shaping species assemblages along elevational gradients, we expect patterns consistent with Fox's guild assembly rule. This rule predicts that on energy‐rich tropical mountains, where both productivity (i.e., resource availability) and species richness decline with elevation (McCain 2009), trait overdispersion should emerge at higher elevations. Such a pattern would indicate that a limited number of species occupy a relatively broad and differentiated niche space, reflecting intensified competitive interactions in resource‐poor environments.

We also expect that increasing taxonomic diversity towards the lowlands is closely associated with denser niche packing, that is, the co‐occurrence of species with similar traits. This pattern may reflect the relatively consistent levels of productivity across adjacent elevational zones in the Mt. Cameroon rainforest (Pellissier et al. 2018; Pigot et al. 2016). To our knowledge, this study is the first of its kind conducted in terrestrial ecosystems of West Africa, and only the second when freshwater systems are also considered (Walsh et al. 2022). Mt. Cameroon hosts the last remaining intact primary forest gradient in the region, extending from lowland areas to the natural treeline. This unique setting offers a rare opportunity to investigate how biotic factors influence faunal community structure in a late‐successional, or climax, stage of forest development.

## 2 Methods

### 2.1 Study Area

We collected the data in the Mount Cameroon National Park along the south‐western slope of the mountain. The location is among the rainiest and wettest places in the world, with annual rainfall above 10,000 mm (Fraser et al. 1998). Precipitation patterns outline two main seasons interspersed with short transition periods (Hall 1973): the wet season (from June to September), the dry season (from December to February), the transition from wet to dry (hereafter wet‐dry; October–November), and the transition from dry to wet (hereafter dry‐wet; March–May). The forest gradient on this slope extends from 350 to approximately 2300 m a.s.l. and can be simply divided into three main zones (see Hořák et al. 2019 for more details on plant community structure): (i) a tropical lowland rainforest (350–900 m a.s.l.) consisting of tall vegetation up to 48 m, scattered understory, and closed canopy; (ii) a mid‐elevation forest (900–1600 m a.s.l.) affected by activities of the African forest elephant ( *Loxodonta cyclotis* ) that create a mosaic landscape of closed and open forest areas, promoting the growth of shrubs and herbs (Fonge et al. 2005; Kamga et al. 2022); (iii) a montane forest (1600‐ca. 2300 m a.s.l.) characterized by persistent fog and cloud cover, patchy canopy with shorter but still large trees (Proctor et al. 2007), and a dense undergrowth consisting of tall herbs and shrubs (Sedláček et al. 2015 and references therein). Although Mt. Cameroon is the highest peak in West Africa (ca. 4040 m a.s.l.), the treeline is located relatively low due to the periodic volcanic activity of the mountain and local hydrological regimes (Proctor et al. 2007; Jacob et al. 2015). The montane forest of this region is known as a ‘hotspot’ of biodiversity across various taxa at the continental level, including birds (Graham et al. 2005; Nana et al. 2014).

### 2.2 Bird Sampling

This represents the most extensive dataset on Mt. Cameroon's avifauna assembled to date. We assembled distributional data for birds collected in 2011 to 2015, 2017 to 2018, and 2021, during all seasons that characterize the local climate: wet‐dry (November), dry (December and February), dry‐wet (March–May), and wet (July–September) seasons. Field surveys were carried out along the whole forest gradient (350–2300 m) across multiple elevational study sites. Here, we summarize elevational distributions for 212 species thus far documented through point counts, mist‐netting, random walks, and by the field expedition of Chmel et al. (2021). Distributional data for birds minimize sampling biases when records are pooled from different sampling techniques (Patterson et al. 1998; Terborgh et al. 1990). In fact, point counts and mist nets offer complementary benefits (Blake and Loiselle 2001). Point counts sample a broad portion of forest bird communities but tend to miss silent species. Mist nets, while not capturing all birds, are effective for detecting inconspicuous ones. Combining both methods allows for more accurate and detailed estimates of birds' elevational ranges than either approach alone. Point counts were carried out during wet‐dry (November 2011, 2021), dry (December 2011, February 2013, December 2015) and wet (July 2021) seasons, using the methodology of Bibby et al. (2000). Point counts in 2011, 2013, and 2015 were performed in the morning (6–10 a.m.) at 16 different points, at each of the following elevations: 350, 650, 1100, 1500, 1850, and 2200 m a.s.l. Each point was at least 150 m apart from another one, and birds were detected through acoustic and visual cues within a 50‐m radius. Bird counts were performed on three different days while swapping the order of points to prevent potential biases due to daytime. During each count, we recorded bird presence in three consecutive 5‐min intervals, and any species detected during these intervals were scored. Similarly, birds were sampled again in July and November 2021 with point counts (Bibby et al. 2000) along the intermediate section of the elevational gradient (900–1700 m). We set a total of 39 sampling points, and each point was visited twice, after a minimum of two days, to improve the quality of the community structure estimate.

We included mist‐netting data, as this method is a vital complement to point counts in forest habitats with dense vegetation (Terborgh 1977). Mist‐netting was carried out during the dry‐wet (March 2012, April–May 2018), wet (August–September 2014, 2017, 2018), wet‐dry (November 2011, 2013–2015, 2017), and dry (December 2011, 2014, 2017–2018) seasons. Birds were mist‐netted using 200 m long line nets (2.5 m tall × 10–15 m long, 16 × 16 mm mesh size) from 6 a.m. to 6 p.m., with checks every 60 min. Mist‐netting was performed at 350, 650, 1100, 1850, 2100, and 2200 m a.s.l.

Random walks within elevation bands were performed from 2011 to 2015 to record the occurrence of rare species. All walks were conducted by three observers for three days to detect rare species not recorded during point count surveys.

Finally, we also incorporated bird records from the field expedition of Chmel et al. (2021). The authors of the study mist‐netted birds at 650 m a.s.l., along the same transect of interest to us, during the wet (August–September) and dry (February) seasons. Embedding the data collected by Chmel et al. (2021) allowed us to gain a more accurate estimate of the elevational distribution of some species (*n* = 15), which from the aforementioned data collections were found only at elevations above 650 m a.s.l.

### 2.3 Species' Elevational Ranges and Bird Assemblages

We assumed that each species was present between the highest and the lowest recorded elevations (i.e., range interpolation). This approach is broadly considered valid for vagile species and ensures methodological consistency, as most studies have assumed range contiguity (Brehm et al. 2007; McCain 2009; Rahbek 1997; Wu et al. 2013). Species' elevational ranges were estimated based on their occurrences in each 200‐m‐wide elevational band between 350 and 2350 m a.s.l. (i.e., 350–549.9 m a.s.l., 550–749.9 m a.s.l., etc.), and all species occurring in each band were regarded as a bird assemblage for analyses. We thus obtained 10 distinct elevational bird assemblages. To estimate the taxonomic diversity along the elevational gradient, we simply counted the number of species occurring within each bird assemblage. Raptors (Accipitridae and Falconidae), swifts (Apodidae), swallows (Hirundinidae), and Palearctic migrants ( *Phylloscopus trochilus* , *P. sibilatrix* ) were excluded from the analyses since we focused only on species breeding in the study area and fairly bound to the forest biotope. Our analyses do not incorporate abundance data, as such information was not consistently available for all species across elevational bands. Finally, we created a list of species for each elevational band.

### 2.4 Functional Traits

Information on avian morphology and life‐history traits was obtained primarily from the Birds of Africa handbook (Brown et al. 2020; Fry and Keith 2020; Keith 2020; Urban et al. 2020) and completed from the dataset available in (Sedláček et al. 2023) and Tobias et al. (2022). For each species, we selected 22 functional traits linked to birds' ability to exploit resources and assumed to mirror the functional role played by species within a community (Sekercioglu 2006; Pellissier et al. 2018; Table S1). These traits can be divided into four broad groups: (i) morphological traits, (ii) diet preferences, (iii) foraging strata, and (iv) feeding strategies. Taken separately, the four trait categories can provide high levels of complementarity in how species exploit the habitat (Adam et al. 2015). Our selection of morphological traits comprised body mass, wing length, tail length, tarsus length, and culmen length. We selected these traits based on the assumption that they are also linked to birds' ecological roles (Miles and Ricklefs 1984; Pigot et al. 2016; Zhang et al. 2020) and may reflect different dimensions of these roles. For diet preferences, we selected six dietary items (invertebrates, vertebrates, fruits, nectar, seeds, and other plant structures such as leaves/stems). For foraging strata, we considered four levels of vegetation stratum that birds explore to search for food (ground, low stratum, middle stratum, and canopy). Finally, we selected seven strategies for feeding (foliage gleaning, bark probing, flycatching, sit‐and‐wait, ground searching, fruit taking, nectar taking). These niche axes represent differences among the behavioral tactics through which resources are exploited. In the literature examined (see above), information on diet, foraging strata, and feeding strategies was provided through descriptions of birds' behavior (i.e., qualitative data). Therefore, for each species, we assigned proportional scores to each niche axis as carried out elsewhere (Pigot et al. 2016; Sedláček et al. 2023). The proportional use of all niche axes within a trait category summed to 1% or 100%. Using this approach, we quantified the proportional use of each niche axis for each species, reflecting the capacity of a species to exploit the structural complexity of the forest.

### 2.5 Phylogenetic Data

From 10,000 phylogenetic trees of birds based on the Hackett backbone (Hackett et al. 2008) and downloaded from http://birdtree.org (Jetz et al. 2012), we generated a 50% majority‐rule consensus tree using the MESQUITE 3.61 program (Maddison and Maddison 2019). Among approaches to reconstruct summary trees, majority rule consensus trees present the most conservative summary of topology, resulting in the inclusion of the fewest incorrect clades (Holder et al. 2008; O'Reilly and Donoghue 2018; Vernygora et al. 2020). The resulting consensus tree contained little polytomy that was resolved using the function *resolve* in the package *RRphylo* (Castiglione et al. 2021). We then used this phylogenetic tree for the phylogenetic analyses.

### 2.6 Evaluation of Functional Trait Space and Functional Diversity

All analyses were performed in R v 4.2.2. (Team 2022) and results were graphed using GraphPad Prism v10 software. We computed functional diversity metrics for each bird assemblage and for each group of functional traits. To evaluate changes in functional diversity in relation to elevation, we first calculated functional traits‐based distances among species using the ‘func.dist()’ function of the *mFD* package (Magneville et al. 2022). Morphological traits‐based distances among species were estimated using the Euclidean distance. Morphological trait values were log‐transformed and scaled (mean = 0 and SD = 1) before computing the Euclidean distance between all pairs of species (Swenson 2014). Subsequently, we carried out PCA analysis using Euclidean traits' distances to generate a trait space within which we computed functional diversity metrics. We assessed the quality of the trait space by computing the mean of absolute deviations (MAD) between trait‐based distances and distances in the Euclidean trait space (Magneville et al. 2022). The lower MAD values are, the better the quality of functional space, meaning that the deviation between trait‐based distance and space‐based distance is minimum (Table S2; Maire et al. 2015).

For each group of ecological traits (i.e., diet, foraging strata, and feeding strategies), we calculated trait distances among species using the Gower distance through the *mFD* package (Gower 1971; Magneville et al. 2022). To calculate functional diversity indices, we first generated a trait space for each set of traits by performing a PCoA analysis using the trait‐based Gower distances. To evaluate the quality of PCoA‐based trait spaces, we assessed the deviation between trait‐based Gower distances and distances in the trait space by calculating the root mean square deviation (RMSD; Maire et al. 2015). The lower the RMSD values are, the better the quality of functional space.

We also built a trait space by gathering both continuous (morphological) and non‐continuous (other ecological) traits. In these cases, the ‘func.dist()’ function enables coding and discriminating different traits as quantitative, nominal, fuzzy, etc. To ensure that the *mFD* package provided an equal contribution of each different group of traits into the trait space, we compared the indices of functional diversity computed using the trait spaces generated through the *mFD* package (see below) with those calculated using the trait space built through the ‘gawdis’ function from the *gawdis* package (de Bello, Botta‐Dukat, et al. 2021). The latter approach has been proposed for properly considering the contribution of different trait categories, handling therefore quantitative, categorical, and other types of traits efficiently (Quimbayo et al. 2024). When using the ‘gawdis’ function, we categorized separately morphological traits from diet and foraging niches and determined the optimal weight for each trait group through an analytical approach, which aims to find a unique mathematical solution for the optimal weight for each group of traits through a system of linear equations (see de Bello, Botta‐Dukát, et al. 2021). We found that correlations between functional diversity metrics computed using the *mFD* package and the corresponding metrics calculated using the gawdis package were high and significant (Figure S2), suggesting that the *mFD* approach provided an equal contribution of all groups of traits. We thus generated a trait space by performing a PCoA analysis using the trait‐based distances calculated with the *mFD* package. The quality of the trait space was evaluated by computing the RMSD (Table S2).

Based on the best functional spaces, we then calculated the functional diversity of each bird assemblage by measuring functional richness (FRic; Cornwell et al. 2006; Villéger et al. 2008), functional mean nearest neighbor distance (FNND; Weiher et al. 1998), and functional evenness (FEve; Villéger et al. 2008) with the R package *mFD*. FRic is a measure of the volume of the functional space occupied by an assemblage. FNND measures the average minimum distance within the functional space of co‐occurring species, reflecting the degree of functional similarity or dissimilarity among species within the community and, thus, how species' traits are densely packed within the assemblage. FEve quantifies the regularity of branch lengths in the minimum spanning tree connecting the species within the functional space (Kraft et al. 2008). Each of these orthogonal functional diversity metrics provides a unique perspective on species' distribution within functional space, accentuating different attributes of functional diversity and collectively enhancing our understanding of community assembly processes (Maglianesi et al. 2015; Villéger et al. 2008).

### 2.7 Evaluation of Phylogenetic Diversity

We calculated phylogenetic diversity for each bird assemblage using two dispersion metrics: mean pairwise distance (MPD) and mean nearest taxon distance (MNTD). MPD is the average of all phylogenetic distances between pairs of species in a sample and, thus, captures information on basal branches of the phylogenetic tree (Montaño‐Centellas et al. 2021; Webb et al. 2002). On the other hand, MNTD is a metric that represents the average minimum phylogenetic distance between species pairs and provides information on the tips of the phylogeny (Montaño‐Centellas et al. 2021; Webb et al. 2002). The latter has previously been found to provide similar information to the classic “Faith's phylogenetic diversity” metric and to respond similarly to changes in species richness and assemblage structure (Mazel et al. 2016). Taken together, the utilization of both MPD and MNTD metrics offers complementary insight into how assembly processes impact the phylogenetic structure of assemblages at different phylogenetic depths (i.e., temporal scales).

To quantify the phylogenetic signal for all morphological traits, we calculated Blomberg's *K* statistic. Specifically, the *K* statistic evaluates the level of phylogenetic signal that would be expected under a Brownian motion model of trait evolution on a given phylogeny (Blomberg et al. 2003). To test the null hypothesis of no signal, we permuted morphological traits across the tips of phylogenies 1000 times. *K* values greater than 1 indicate significant phylogenetic signal and conservatism of traits (Kembel et al. 2010). To quantify MPD and MNTD, and the phylogenetic signal of morphological traits, we used functions of the *picante* package (Kembel et al. 2010).

### 2.8 Determinants of Community Assembly

In order to explore the effect of environmental filtering, limiting similarity, and guild assembly rule on community assembly along the elevational gradient, we tested whether observed functional and phylogenetic diversities were different from expected by chance. For this purpose, we generated null models by randomizing traits and phylogeny tip labels 500 times across the observed trait data matrices and phylogeny, respectively. We preferred this approach over randomizing the elevational distribution of species across the observed community data matrix, as this allowed us to control for species richness and range contiguity, reducing the type I error probability (Pigot et al. 2016; Swenson 2014). We then estimated the deviation of each observed functional and phylogenetic metric from the average of their null distribution and standardized the values obtained to allow comparisons among assemblages (standardized effect size, SES; Swenson 2014). All expressed as follows:

$$\mathrm{SES}=\frac{\text{Observed}-\bar{X}}{\mathrm{SD}\left(X\right)}$$

where Observed is the observed value of a given functional or phylogenetic metric, $\bar{X}$ is the average of the null distribution of the given functional or phylogenetic metric, and SD(*X*) is the standard deviation of the null distribution of the given functional or phylogenetic metric. Positive SES values of FRic and FNND were interpreted as functional overdispersion, whereas negative SES values were interpreted as functionally clustered assemblages. Positive SES values of FEve were interpreted as a higher regularity of trait spacing than expected by random, whereas negative SES values were regarded as a lower regularity of trait spacing than expected by random. Positive SES values of MPD and MNTD were interpreted as phylogenetic overdispersion, whereas negative SES values were interpreted as phylogenetic clustering (or clustering). We deemed SES values to be statistically significant (i.e., indicating a deterministic signal) if the observed value of a given functional or phylogenetic metric fell outside the 2.5%–97.5% quantiles of the null distribution.

### 2.9 Changes in Functional and Phylogenetic Diversity Along the Elevational Gradient

In order to determine abrupt changes in observed and SES patterns across the elevations, we used segmented regressions (‘segmented’ function, ‘*segmented*’ package; Muggeo 2008) in R. While it is generally possible to have multiple changes or breakpoints, we assumed a maximum of one change for each relationship for easy interpretability with a narrow set of data points available (only 10). Our choice did not compromise goodness of fit and, overall, explained well the relationships (mean *R* ^2^ = 0.88). The segmented regression seeks two different lines in a changing regression between the response and explanatory variables, identifying in our case where significant shifts in the observed and SES metrics occurred along the elevational gradient. We employed the Davies test to evaluate whether the two slopes were significantly different from each other (Cooper et al. 2021; Davies 2002). If no changes were identified because Davies test was not significant at *p* < 0.05, we considered the relationship to be linear. Although elevation was categorized into discrete elevational bands, we modeled elevation as a continuous variable by using the lower elevational limit of each respective elevational band in the segmented regression.

### 2.10 Quantifying Relative Contributions of Niche Packing and Expansion to the Increase in Species Richness

In order to determine the relative contributions of niche packing and expansion to the increase in species richness between adjacent elevational bands, we used the niche packing “flexible” metric developed by Pigot et al. (2016). Through this algorithm, when comparing a species‐richer assemblage (A_1_) with a species‐poorer assemblage (A_2_), we iteratively removed unique species from A_1_ (i.e., absent in A_2_) that contributed the most to the functional volume until its size was equal to or lower than that of A_2_. The percentage of unique species remaining in A_1_ after this sequence represents the increase in species richness due to niche packing, whereas the percentage of unique species removed at the end of the simulation represents the increase in species richness due to niche expansion.

## 3 Results

### 3.1 Taxonomic, Functional and Phylogenetic Diversity Gradients

Altogether, 212 avian species were recorded over 10 years of field surveys. Taxonomic diversity decreases almost monotonically with increasing elevation, following a low elevation hump pattern (Figure S1b). All functional guilds are represented along the elevational gradient, indicating that changes in species richness are due to variations in richness within guilds (Figure S1a,c,d). The species richness gradient is dominated by invertivores that glean prey from foliage, followed by birds harvesting fruits (Figure S1a,d). Barely, bark probers contribute to changes in species richness, and the least contribution is given by folivores (Figure S1a). Variations in species richness are partitioned equally across guilds using different vegetation strata, with the only exception being a slight increase in the richness of “ground users” with elevation at the expense of species foraging in the canopy (Figure S1c).

Except for dietary traits, observed FRic decreases linearly with elevation (Morphological traits: *t* = −9.28, *p* < 0.001; Foraging strata: *t* = −2.77, *p* < 0.05; Feeding strategies: −8.57, *p* < 0.001; All traits: *t* = −2.64, *p* < 0.05; Figure S3a–e; Table S4). In contrast, segmented models show a positive trend between observed FNND and elevation for each type of trait, though with different breakpoints (Figure S3f–j; Table S4). As shown in Figure S3, observed FEve based on diet, feeding strategies, and all traits combined, increases with elevation (Dietary traits: *t* = 7.97, *p* < 0.001; Feeding strategies: *t* = 6.50, *p* < 0.001; All traits: *t* = 5.23, *p* < 0.001). Based on morphology, FEve increases significantly from the lowest elevation up to ~1950 m (*t* = 4.56, *p* < 0.05) and then decreases towards the upper end of the elevational gradient (Table S4, Figure S3k). FEve based on foraging strata also shows a positive trend against elevation, but only from ~600 m.

All morphological traits have significant phylogenetic signal (*K* > 1, *p* < 0.01; Table S3), suggesting that phylogenetic distances can be considered as a proxy for differences in morphological characteristics for birds in this study.

Observed MPD shows a negative but non‐significant linear trend with elevation (Figure S4a; Table S6). On the other hand, the segmented regression model shows a positive relationship between observed MNTD and elevation (*t* = 2.97, *p* < 0.05), which becomes steeper above ca. 1950 m (*t* = 3.40), although there is only one data point beyond this threshold (Figure S4b; Table S6).

### 3.2 Differences From Random Patterns of Functional and Phylogenetic Diversity

**FIGURE 2:**
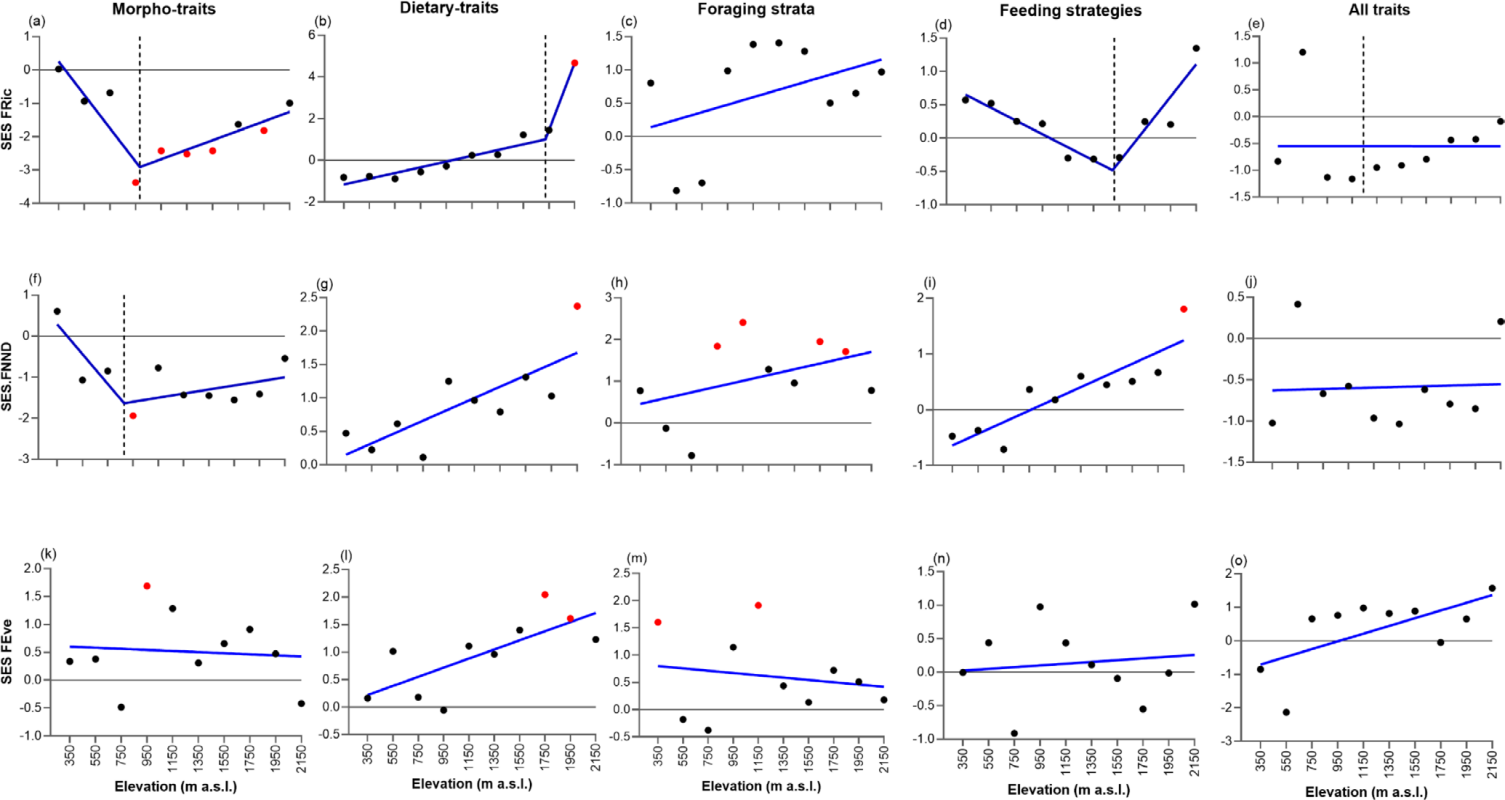
Functional diversity patterns along the elevational gradient of Mt. Cameroon. Columns are sets of traits and rows are different metrics after controlling for species richness: Functional richness (SES.FRic), functional nearest neighbor distance (SES.FNND), and functional evenness (SES.FEve). Blue lines represent the fit of segmented and linear regressions. Vertical dashed lines show the elevation where a statistically significant shift in the trend occurred. Horizontal solid lines at *y* = 0 indicate null predictions. Therefore, red dots above 0 indicate significant functional overdispersion (*p* < 0.05) and below 0 indicate significant functional clustering. Note that in panel (l), the dashed line is not visible, as it coincides with the abscissa.

Accounting for species richness and range contiguity, SES.FRic based on morphological traits was significantly low between 950 and 1550 m (from −3.37 to −2.42), and at 1950 m (−1.81; Figure 2a). SES.FRic, based on dietary traits, increased significantly with elevation and showed that observed values of functional richness were significantly higher than expected at random at 2150 m (4.67). None of the observed functional richness values were significantly different from random when considering traits related to vegetation strata usage, foraging strategies, and all combined traits (Figure 2c–e).

SES.FNND based on morphological traits was −1.94 at 950 m, indicating functional clustering at this elevation (Figure 2f). All SES.FNND values related to dietary traits were positive, indicating thus consistent functional overdispersion, especially at 2150 m (2.37; Figure 2g), as in the case of the SES.FNND related to foraging strategies (1.80; Figure 2i). SES.FNND for traits related to vegetation strata indicated significant functional overdispersion at mid (950 m: 1.84; 1150 m: 2.41) and high elevations (1750 m: 1.95; 1950 m: 1.71) (Figure 2h).

When examining effect sizes of trait evenness (SES.FEve), we found that assemblages were more evenly packed than expected at random at 950 m (1.68) and from 1750 to 1950 m (2.05–1.61) based on morphology and diet, respectively (Figure 2k,l). Furthermore, higher values of observed functional evenness than expected at random were found in the lowlands (350 m: 1.6) and at mid‐elevations (1150 m: 1.91) for traits related to the usage of vegetation strata (Figure 2m).

**FIGURE 3:**
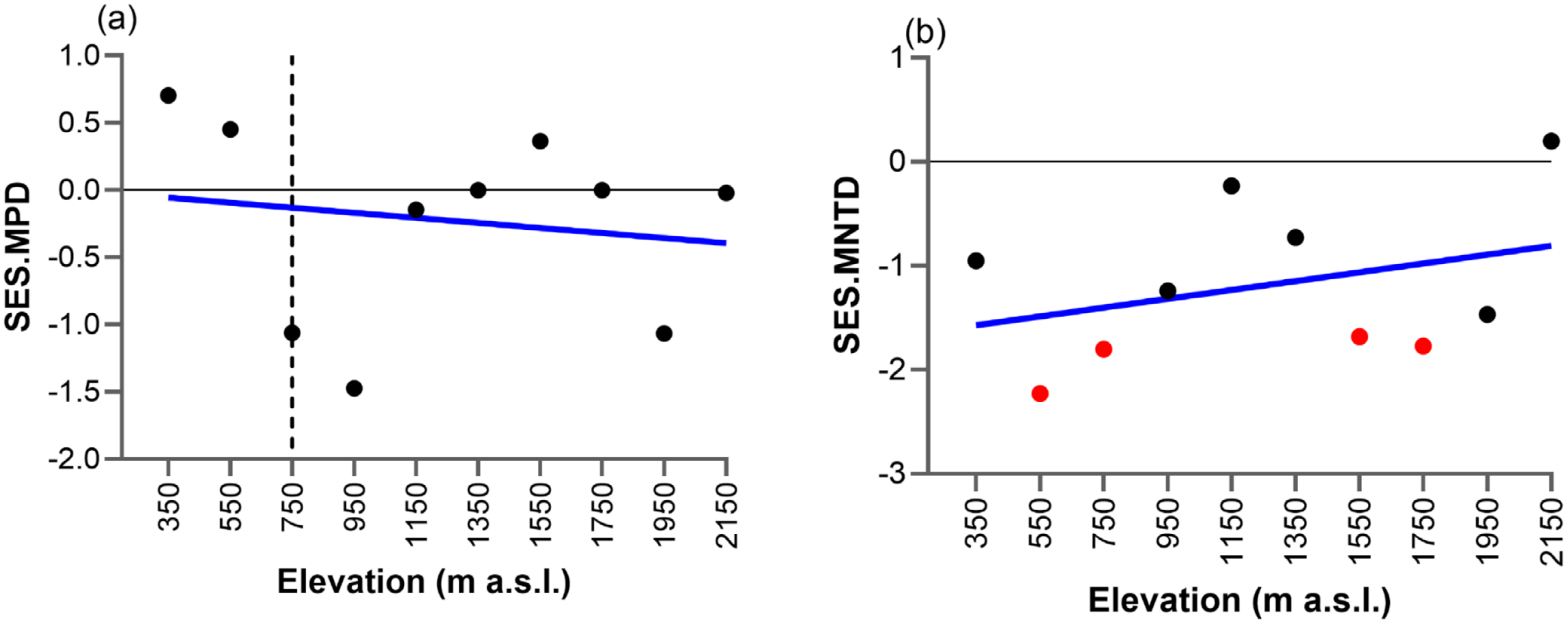
Relationship between elevation and effect sizes of phylogenetic diversity metrics (SES) measured using mean pairwise distance (MPD) and mean nearest taxon distance (MNTD). Blue lines = segmented and linear regression models; dashed vertical lines = breakpoints where shifts in the trends occurred; solid horizontal line = null predictions (*y* = 0). Dots reflect phylogenetic overdispersion (values > 0) or clustering (values < 0). Red dots indicate significant overdispersion‐ or clustering compared to null model simulations.

Across elevations, the basal phylogenetic structure of the assemblages (SES.MPD) was consistent with that expected at random (Figure 3a). Conversely, significant negative SES.MNTD values at low (550 m: −2.23; 750 m: −1.80) and high elevations (1550 m: −1.68; 1750 m: −1.77) indicated significant terminal phylogenetic clustering, suggesting thus the co‐occurrence of closely related species (Figure 3b).

### 3.3 Trends in Standardized Effect Size of Functional and Phylogenetic Diversity Along the Elevational Gradient

Based on morphological traits, we observed a significant decrease in SES.FRic between 350 and ~950 m (Figure 2a; Table S5). The segmented regression model indicated a similar negative relationship between elevation and SES.FNND, although the slope was not significant (Figure 2f; Table S5). Analysis of dietary traits showed, in general, a positive trend between elevation and all metrics (Figure 2b,g,l). Thus, SES.FRic increased from the lowest elevation up to ~1900 m (*t* = 6.24, *p* < 0.001) and then increased more steeply with elevation (*t* = 7.16), whereas SES.FNND and SES.FEve showed a positive significant linear relationship with elevation (SES.FNND: *t* = 3.60, *p* < 0.001; SES.FEve: *t* = 3.08, *p* < 0.05). All standardized functional diversity metrics based on foraging strata were best fit by linear relationships with elevation (Figure 2c,h,m). SES.FRic and SES.FNND both showed a positive but non‐significant trend (SES.FRic: *t* = 1.34, *p* > 0.05; SES.FNND: *t* = 1.33, *p* > 0.05), whereas SES.FEve showed a negative, non‐significant trend (*t* = −0.49, *p* > 0.05). Analysis of traits related to foraging strategies revealed that SES.FNND increased linearly and significantly with elevation (Figure 2i; *t* = 5.30, *p* < 0.001). On the other hand, SES.FRic showed a positive, significant relationship with elevation only above ~1500 m (Figure 2d; *t* = 4.81, 95% CI = 0.0012–0.0037). When analyzing all traits together, standardized functional diversity metrics showed no significant trend with elevation (Figure 2e–o).

None of SES. MPD and SES.MNTD had significant relationships with elevation (Figure 3). SES.MPD showed a weak, negative linear relationship with elevation (*t* = −0.45, *p* > 0.05), whereas SES.MNTD displayed a weak positive trend with elevation (*t* = 1.01, *p* > 0.05).

### 3.4 Relative Contributions of Niche Packing and Niche Expansion to Increasing Species Richness

**FIGURE 4:**
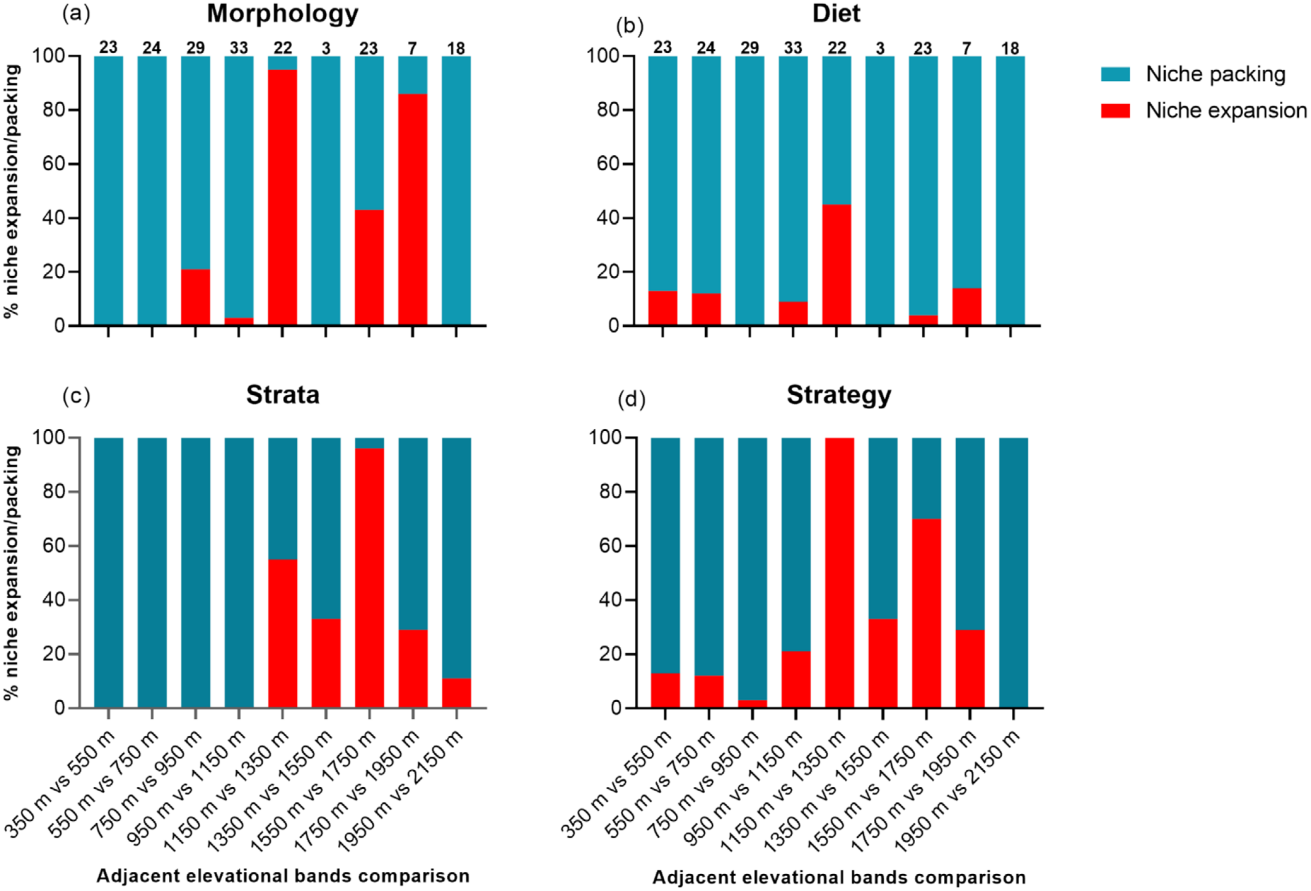
Niche packing and expansion along the Mt. Cameroon elevational gradient calculated with different sets of traits: Morphology (a), diet (b), foraging strata (c), feeding strategies (d). The relative importance of niche packing (blue) to changes in species richness across adjacent elevational bands is expressed as the percentage of species unique to a species‐rich assemblage accommodated within the functional volume of a species‐poor assemblage. Conversely, species unique to a species‐rich assemblage that do not fit within the functional volume of a species‐poor assemblage contribute to niche expansion (red). Numbers above bars in panels (a) and (b) indicate the number of unique species between an adjacent elevational band pair.

When estimating the relative contributions of niche packing and niche expansion among bird assemblages using different sets of traits, we found that most of the additional species in the lowlands were packed within the functional volume occupied by adjacent species‐poorer assemblages (Figure 4). Particularly, niche packing was dominant in the lowlands up to 1150 m for traits related to foraging strata (Figure 4c). On the other hand, niche expansion was relatively high at high and mid‐elevations (1150–1350 m), especially when calculated using morphological traits, foraging strategies, and all combined traits (Figure 4a,d; Figure S5). The highest niche expansion for dietary traits was also recorded when comparing the 1150‐m‐assemblage with the 1350‐m‐assemblage. The majority of additional species at around 1550 m occurred outside the functional volume of the 1750‐m assemblage when analyzing foraging strata, feeding strategies, and all traits combined (Figure 4c,d; Figure S5).

## 4 Discussion

### 4.1 Assembly Processes Along the Elevational Gradient

When investigating the structure of the functional space according to different traits, we found only partial support for the role of environmental filtering in shaping bird assemblages. SES.FRic, based on morphological traits, significantly declined from 350 to 950 m, with the 950‐m assemblage showing the lowest negative SES.FRic value. This does not align with our expectation of a trait filtering more dominant at high elevations and driven by climatic variables, despite steady declines in temperature and precipitation along the elevational gradient (Maicher et al. 2020). While climatic factors such as precipitation and temperature can directly influence taxonomic diversity (Peters et al. 2016), their effects on functional richness, as measured by morphological traits, appear limited (Hanz et al. 2019). It is possible that species retain their morphological attributes while evolving physiological traits to cope with harsher climatic conditions (Londoño et al. 2017). Unfortunately, we could not integrate physiological traits related to climatic gradients since such traits are unavailable for many species in our study. In addition, we did not observe further evidence of environmental filtering through SES.FRic calculated from other types of traits. For example, SES.FRic based on diet showed a positive trend with increasing elevation until it was greater than expected by chance at the treeline. Similarly, in contrast to environmental filtering expectations, we observed increasing SES.FNND towards higher elevations. This indicates that species at the periphery of the dietary space were not selectively filtered out as elevation increased and suggests that the effect of environmental filtering might be weak due to mild local climatic conditions and relatively high resource availability (due to high environmental productivity and low treeline) in the upper zones of the elevational gradient. Especially with regard to the latter, previous research on Mt. Kilimanjaro and Ecuadorian Andes found that functional diversity of bird assemblages decreased significantly with increasing elevation, a pattern mainly associated with gradients in resource availability (Hanz et al. 2019). Even though the two elevational gradients in that study present a similar elevational span to that of Mt. Cameroon (*c*. 2000 m), the latter has a lower treeline (Mt. Cameroon: 2300 m; Mt. Kilimanjaro: 3060 m; Ecuadorian Andes: 2898 m). This supports the idea that climatic harshness along our sampled gradient was not strong enough to constrain the resource spectrum extensively, reducing the effect of filtering on ecological guilds.

In contrast to other recent studies on tropical bird communities (Boyce et al. 2019; Hanz et al. 2019; Montaño‐Centellas et al. 2021), we did not find evidence of functional overdispersion at low elevations. We observed decreasing trait distances among co‐occurring species compared with null expectations (SES.FNND) towards lower elevations for most sets of traits (i.e., dietary traits, foraging strata, and feeding strategies). Such results contrast with the predictions of the limiting similarity theory (Cavender‐Bares et al. 2009; MacArthur and Levins 1967), downplaying the role of competition in less abiotically stressful elevations. However, competition can also drive functional clustering when competitive dominance is associated with specific traits, leading to trait convergence among the remaining species (HilleRisLambers et al. 2012; Mayfield and Levine 2010; Perronne et al. 2017). According to this expectation, interspecific competition could cause lowland species assemblages to exclude highland species, resulting in lower elevations being dominated by lowland specialists with similar functional traits. However, our analysis of niche packing/expansion contribution suggests that functional clustering in the lowlands could arise because of a high degree of niche overlap in environments with high resource capacity, which is typical of lowland tropical forests (Ferger et al. 2014; Klopfer and MacArthur 1961; Pellissier et al. 2018). Indeed, when quantifying directly niche packing and expansion contributions to taxonomic diversity, we found that the species richness gradient of Mt. Cameroon was mainly associated with dense trait packing. This appears particularly evident in the pairwise comparisons between lowland assemblages, where in some cases niche packing contributed up to 100% to increases in richness. Our results are consistent with previous studies on birds along tropical (Pigot et al. 2016) as well as temperate (Schumm et al. 2020) elevational gradients, where niche packing was the dominant pattern underlying diversity gradients. Similarly, Chakravarty et al. (2023) showed that the high bat diversity at low elevations in the Himalayas was driven by niche packing, suggesting a generality of the rule across different taxa. Moreover, mid‐ and high‐elevation assemblages exhibited significant overdispersion in traits related to vegetation strata. This finding might be attributed to the gradient in vegetation structure along the forested slope. Mid‐elevation and montane forests on Mt. Cameroon exhibit greater herb layer coverage due to increased canopy openness (Hořák et al. 2019). In some sense, although reduced complexity of vegetation structure negatively correlates with bird richness (Hořák et al. 2019; Jankowski et al. 2013; Kamga et al. 2022), a slight decrease in forest density may benefit certain species by offering nesting sites, shelter, and feeding substrates. One example is ground‐ and low‐stratum feeders, such as *Estrilda nonnula* and *Cisticola chubbi* , which might exploit the herbaceous cover for foraging and predator avoidance. Hence, the increase in trait distances with decreasing vegetation complexity suggests that the loss of forest structure may allow the occurrence of certain functional groups of birds. Previously, the vegetation structure of Mt. Cameroon was not identified as a significant predictor of avian functional diversity (Hořák et al. 2019). In contrast to this study, the authors assessed functional diversity solely based on morphological traits, which likely did not capture the full extent of ecological diversification.

Other works pointed out that signals of abiotic filtering can emerge when using abundance‐based metrics (Freilich and Connolly 2015; Montaño‐Centellas et al. 2021). In our context, including species abundances might have changed the outcome of the analysis. The highlands of the Cameroonian mountains are known to support high local densities of forest bird species (Ferenc et al. 2018; Reif et al. 2006). We do not exclude that, although functionally redundant species disappear towards higher altitudes, the free ecological space could be compensated by individuals of the remaining species, potentially reducing SES.FNND values. For example, nectarivores, which show increased abundance with elevation despite decreasing taxonomic diversity on Mt. Cameroon (Sedláček et al. 2023), may contribute to this compensation. However, it is possible that the implementation of abundance data would only lead to different results from a quantitative, rather than qualitative standpoint. In other words, the use of a presence‐absence matrix would not reveal discordant information from that provided by an abundance matrix. Moreover, presence‐absence data may better capture the effects of environmental filtering because they directly reflect whether a species is adapted to a particular habitat (Gutiérrez et al. 2013). Future work testing the differences in outcomes between abundance and presence‐absence data on our species could provide valuable insights, especially considering that abundance data may more effectively capture the effects of biotic interactions on community assembly (Brian and Aldridge 2021).

The limiting similarity hypothesis predicts that resource limitation drives competitive interactions, leading to a more regular spacing of species within the functional space (Fleming 1979; MacArthur and Levins 1967). Therefore, we expected an increased regularity in functional distances (SES.FEve) among co‐occurring species in the lowlands, where competition is likely more intense (Graham et al. 2009). Surprisingly, we found that the regularity of distances among species within lowland assemblages was higher than expected at random only at 350 m a.s.l. for traits related to vegetation strata usage. Limits to phenotypic similarity did not constrain bird richness in the lowlands, as functional evenness based on morphological traits was significantly higher than random expectations only at 950 m a.s.l. Notably, we detected signals of limiting similarity in diet traits at high elevations, suggesting that interspecific competition for reduced amounts of food resources in montane forests might constrain local taxonomic diversity. While the mechanisms behind the observed patterns remain speculative, our results highlight the importance of exploring multiple trait dimensions when testing the limiting similarity hypothesis, as competition may operate through various aspects of ecological space.

We found partial support for Fox's guild assembly rule (Brown et al. 2000; Fox 1987). Consistent with this rule, high‐elevation, low‐richness assemblages showed greater dietary and foraging mode trait distances (SES.FNND) than low‐elevation, high‐richness assemblages. Moreover, the assemblage at the treeline (*c*. 2250 m a.s.l.) was significantly overdispersed, showing observed functional nearest neighbor distances substantially higher than expected at random. Similarly, Kohli et al. (2021) also found some support for Fox's hypothesis in small mammals, reporting overdispersed species‐poor assemblages at high elevations in the Snake Range and Ruby Mountains, where yearly temperatures are very low. This hypothesis, however, received no support in other studies. For instance, Feeley (2003) found no evidence of the guild rule within Venezuelan islands, as avian communities were unbalanced, with insectivores being over‐represented. The insectivorous guild is, however, frequently the most abundant one in tropical forests (Blake and Loiselle 2000; Sam et al. 2017); it holds for Mt. Cameroon as well (Sedláček et al. 2023). Thus, more nuanced analyses of community structure may be necessary to find stronger support for the guild assembly rule. Nevertheless, we acknowledge that the SES.FNND patterns observed in our study do not directly demonstrate guild‐based assembly. The increase in SES.FNND estimated from diet and foraging mode traits with increasing elevation may be the result of species absence due to extinction filter (Williams et al. 2009). We found that high‐elevation assemblages on Mt. Cameroon are rather depauperate (in terms of species numbers) compared with lowland assemblages. Such a reduced taxonomic diversity on this mountain is presumably due to both high extinction rates in smaller areas of suitable habitats and low colonization rates posed by harsh abiotic barriers of lowland habitats over the Pleistocene (Ferenc et al. 2016; Reif et al. 2006). Functional diversity may be less influenced by extinction and colonization rates than taxonomic diversity, as high trait redundancy can lead to functional overdispersion even when species richness is kept low. However, this hypothesis requires further comparative studies for confirmation.

### 4.2 Contrasting Biodiversity Facets

We found a mismatch among taxonomic, functional, and phylogenetic diversities along the elevational gradient, providing further evidence that different factors shape distinct facets of biodiversity, as already corroborated in existing literature (Aguirre et al. 2016; Stevens et al. 2013; Stevens and Gavilanez 2015). Specifically, we observed considerable differences between taxonomic diversity and the functional mean nearest neighbor distance (FNND). While taxonomic diversity decreased with elevation, FNND exhibited an increasing trend. This indicates that the relatively few species at high elevations are functionally divergent, whereas lowland assemblages are rich in functionally redundant species. As discussed above, this outcome is in line with the niche packing theory and suggests that trends in taxonomic diversity and functional trait distances may be influenced by shared drivers (e.g., resources availability; McClain et al. 2018) but in the opposite direction.

We also examined variations in the phylogenetic structure of bird assemblages along the elevational gradient. Although most studies report decreasing phylogenetic diversity with elevation (Dehling et al. 2014; Hanz et al. 2019; Montaño‐Centellas et al. 2021), we found dissimilar patterns between taxonomic and phylogenetic diversity metrics. Notably, observed terminal phylogenetic distances (MNTD) showed a positive trend up to ~1950 m. Moreover, signals of terminal phylogenetic clustering (i.e., negative SES.MNTD values) at both low and high elevations indicate the co‐occurrence of closely related species in these elevational zones. This clustering could be attributed to recent radiations of specific clades driven by climatic fluctuations during the late Tertiary and Quaternary, which subjected Afromontane forests to cycles of expansion and retraction (Elenga et al. 2000; Newton 2003; Voelker et al. 2010). These dynamics could have favored isolation and, consequently, radiations of forest‐dwelling clades on Mt. Cameroon (Fjeldsaå and Lovett 1997; Reif et al. 2006). Overall, our results suggest recent radiations of particular clades at the edges of the elevational gradient of Mt. Cameroon, which leads to terminal phylogenetic clustering.

The trend in mean phylogenetic distance (MPD) was negative and showed no clear signals of deterministic processes acting on the basal structure of phylogenies when compared with the random expectations (SES.MPD). We argue that this random phylogenetic pattern may reflect the balanced distribution of evolutionarily distinct subclades (e.g., Nectariniidae, Estrildidae, Pycnonotidae) across elevations, with comparable species numbers in the assemblages.

We found no clear congruence between functional and phylogenetic diversity patterns along elevation, contrasting with recent studies (He et al. 2022; Liu et al. 2023; Sun et al. 2023). However, observed functional nearest neighbor distances (FNND) and mean nearest taxonomic distances (MNTD) both exhibited a positive trend with elevation. In some cases, SES.MNTD and SES.FNND highlighted divergent functional and phylogenetic assembly structures. For instance, at higher elevations, SES.MNTD indicated phylogenetic clustering, while SES.FNND, based on diet and foraging strategies, suggested functional overdispersion. This result implies that close relatives did not necessarily share similar functional characteristics. Traits related to diet and foraging characteristics may possess a degree of flexibility and have potentially evolved differently within clades to exploit varying local resources so that species do not have to compete closely (Burin et al. 2016; Kissling et al. 2012). Accordingly, Jarzyna et al. (2021) reported that functional and phylogenetic structures become more incongruent with increasing elevation in the tropics. Furthermore, this mismatch may arise when certain functional traits are subject to strong stabilizing selection or influenced by competitive interactions within lineages (E‐Vojtkó et al. 2023; Prinzing et al. 2008). Our results seem to provide additional evidence that phylogenetic diversity cannot be considered a reliable surrogate for functional diversity and reinforce the idea that these two biodiversity facets can provide complementary insights into ecological processes structuring community assembly (Mazel et al. 2017, 2018; Montaño‐Centellas et al. 2023; Tucker et al. 2018).

## 5 Conclusions

To our knowledge, this is the first study combining different facets of diversity along an Afrotropical elevational gradient to understand processes behind avian community assembly. Our measurements of functional diversity, assessed through multiple metrics, did not detect strong signals of abiotic filtering, likely due to the highly productive yet relatively short forest gradient of Mt. Cameroon. Our findings suggest that low elevations, known to be abiotically benign and rich in resources (Hanz et al. 2019), appear to host a great number of functionally redundant species, potentially downplaying the role of interspecific competition. At the same time, we showed that the increasing number of species towards lower elevations is associated with denser niche packing. We revealed increasing null‐model corrected effect sizes of functional distances among co‐occurring species along the elevational gradient when examining traits related to diet and foraging strategies, providing partial support for Fox's hypothesis. Building on previous studies (Ferenc et al. 2016; Sedláček et al. 2023), the availability of resources in the highland forest likely remains sufficiently high to support high population densities among the few present functionally divergent species. Finally, phylogenetic terminal clustering and random‐consistent basal phylogenetic structures at low and high elevations suggest explosive radiation within multiple major subclades in the lowlands and highlands, probably caused by montane forest expansion and contraction throughout climatic cycles in the late Tertiary and Quaternary (Newton 2003; Reif et al. 2006). This implies the existence of evolutionary distinct lowland and highland faunas.

## Author Contributions

**Riccardo Pernice:** conceptualization (equal), data curation (lead), formal analysis (lead), writing – original draft (lead), writing – review and editing (lead). **Ondřej Sedláček:** investigation (equal), writing – review and editing (supporting). **Tomáš Albrecht:** funding acquisition (supporting), investigation (equal), writing – review and editing (supporting). **Oldřich Tomášek:** investigation (equal), writing – review and editing (supporting). **Ondřej Kauzál:** investigation (equal), writing – review and editing (supporting). **Tereza Kauzálová:** investigation (equal), writing – review and editing (supporting). **Francis Njie Motombi:** investigation (equal), writing – review and editing (supporting). **Francis Luma Ewome:** investigation (equal), writing – review and editing (supporting). **Michal Ferenc:** data curation (supporting), investigation (supporting), writing – review and editing (supporting). **Kryštof Chmel:** investigation (equal), writing – review and editing (supporting). **Jiří Mlíkovský:** investigation (equal), writing – review and editing (supporting). **Jan Riegert:** funding acquisition (supporting), investigation (equal), writing – review and editing (supporting). **Solange Mekuate Kamga:** investigation (equal), writing – review and editing (supporting). **David Hořák:** conceptualization (equal), funding acquisition (lead), investigation (supporting), writing – original draft (supporting), writing – review and editing (supporting).

## Conflicts of Interest

The authors declare no conflicts of interest.

## Supporting information


**Data S1:** ece372065‐sup‐0001‐Supinfo.docx.

## Data Availability

Dataset and R code used in this study are available in the following repository: https://doi.org/10.5061/dryad.tdz08kq79.
