# Supplementary material for: Functional and Phylogenetic Structure of Forest Bird Assemblages Along an Afrotropical Elevational Gradient
Source: Ecol Evol. 2025 Aug 27;15(9):e72065. doi: 10.1002/ece3.72065 (PMC12391590; doi:10.1002/ece3.72065)
Supplement: Supplementary file 1 — Data S1: ece372065‐sup‐0001‐Supinfo.docx. [file ECE3-15-e72065-s001.docx]

Ecology and Evolution

SUPPORTING INFORMATION

Functional and phylogenetic structure of forest bird assemblages along an Afrotropical elevational gradient

Riccardo Pernice, Ondřej Sedláček, Tomáš Albrecht, Oldřich Tomášek, Ondřej Kauzál, Tereza Kauzálová, Francis Njie Motombi, Francis Luma Ewome, Michal Ferenc, Kryštof Chmel, Jiří Mlíkovský, Jan Riegert, Solange Mekuate Kamga, David Hořák

**Appendix S1**

We estimated the proportions of species in each assemblage that utilize specific diets, foraging modes, and feeding strata across all elevations (Figure S1). Since species often utilize multiple dimensions within their niche (i.e., different types of food), we characterized each species based on the proportions of each niche dimension it uses (Sedláček et al., 2023). For instance, if a species derives 30% of its diet from invertebrates, we assigned a value of 30 to this category. The sum of all categories at a given elevation equals 100%.

**
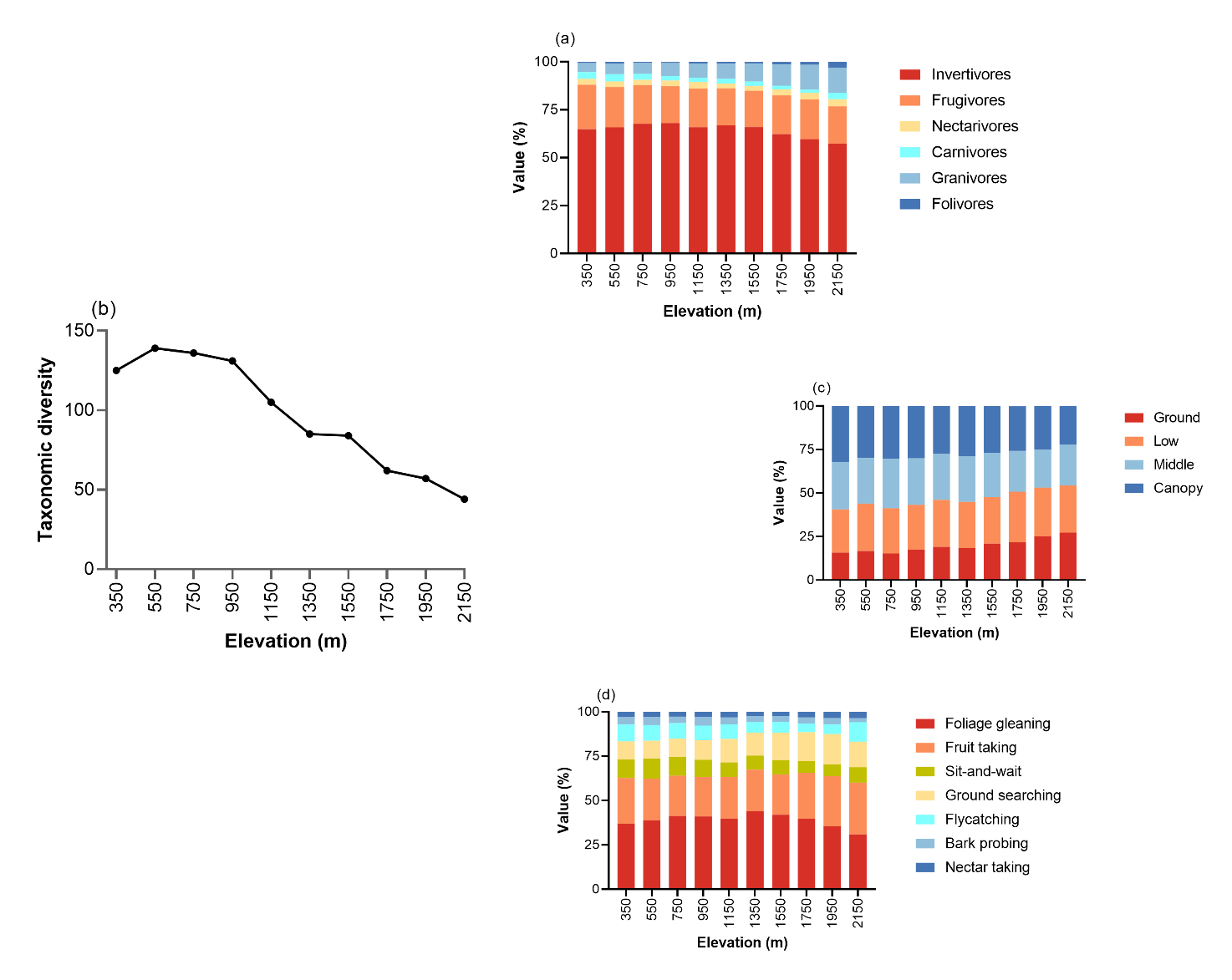
**

**FIGURE S1.** Assemblage taxonomic diversity across elevations (b) and proportional contribution of each functional guild to the observed elevational species richness pattern (a, c, d).

**Appendix S2**

In order to create a multidimensional functional space in which functional diversity metrics were calculated, we carried out a PCoA using trait-based distances. Since the number of PCoA axes may affect functional diversity indices, it is crucial to determine the best functional trait space. We thus estimated the quality of functional spaces according to the deviation between trait-based distances and distances within the functional space (Maire et al., 2015) by using functions in *mFD* package (Magneville et al., 2022). For continuous traits, we calculated the mean absolute deviation (mad) between trait-based distances and distances within the functional space, whereas, for fuzzy traits, we computed the root mean square deviation (rmsd). The number of PCoA axes retained that best represent the functional space, for each set of traits, is shown in Table S2.

We then calculated functional diversity and phylogenetic diversity of each bird assemblage within the best functional spaces. Specifically, we estimated three facets of the functional diversity: (i) functional richness (FRic; Cornwell et al. 2006, Villéger et al. 2008), (ii) functional mean nearest neighbor distance (Weiher et al. 1998), and (iii) functional evenness (Villéger et al. 2008). Before calculating phylogenetic diversity metrics, we assessed whether there was phylogenetic signal in species’ morphological traits. We quantified the phylogenetic signal in morphology of all birds by using the *K* statistic proposed by Blomberg et al. (2003) (Table S3). To assess phylogenetic diversity, we measured mean pairwise dissimilarity (MPD) and mean nearest taxon distance (MNTD) (Kembel et al., 2010; Montaño-Centellas et al., 2021; Webb et al., 2002). Finally, we analyzed how functional and phylogenetic metrics were related to the elevational gradient (Figure S3, S4). We fitted simple linear regression model to each functional and phylogenetic metrics with elevation as the explanatory variable. To assess potential breakpoints of linear relationships in the patterns of functional and phylogenetic metrics along the elevation, we fitted segmented regressions using the R package *segmented* (Table S3-5; Muggeo and Muggeo 2017).

**TABLE S1** Functional traits estimated for birds within assemblages along elevation of Mt. Cameroon.

| **Functional trait** | **Functional trait description** | **Trait category** | **Trait type** |
| --- | --- | --- | --- |
| Body mass | Mean body mass (g) | Morphology | Continuous |
| Wing length | Mean wing length (mm), measured from the carpal joint to the tip of the longest primary | Morphology | Continuous |
| Tail length | Mean tail length (mm), measured from the base to the tip of the longest tail feathers | Morphology | Continuous |
| Tarsus length | Mean tarsus length (mm) | Morphology | Continuous |
| Culmen | Mean bill length (mm) | Morphology | Continuous |
| Invertebrates | Percentage of diet consisting of insects and other invertebrates | Diet | Fuzzy trait |
| Vertebrates | Percentage of diet consisting of vertebrates (small reptiles, mammals, etc.) | Diet | Fuzzy trait |
| Fruits | Percentage of diet consisting of fruits | Diet | Fuzzy trait |
| Nectar | Percentage of diet consisting of nectar | Diet | Fuzzy trait |
| Seeds | Percentage of diet consisting of seeds | Diet | Fuzzy trait |
| Other plant structures | Percentage of diet consisting of other plant materials (leaves, stem, etc.) | Diet | Fuzzy trait |
| Ground | Percentage of use of ground feeding stratum | Foraging stratum | Fuzzy trait |
| Low stratum | Percentage of use of low vegetation stratum | Foraging stratum | Fuzzy trait |
| Middle stratum | Percentage of use of middle vegetation stratum | Foraging stratum | Fuzzy trait |
| Canopy | Percentage of use of canopy feeding stratum | Foraging stratum | Fuzzy trait |
| Foliage gleaning | Percentage of use of foliage gleaning tactic for collecting food items | Feeding strategy | Fuzzy trait |
| Bark probing | Percentage of use of bark probing tactic for collecting food items (e.g. from trunks, branches, etc.) | Feeding strategy | Fuzzy trait |
| Flycatching | Percentage of use of flycatching tactic for collecting food items (i.e. birds taking preys in air) | Feeding strategy | Fuzzy trait |
| Sit-and-wait | Percentage of use of sit-and-wait tactic for collecting food items (i.e. species taking a prey in air or from any substrate surface after remaining stationary for long time | Feeding strategy | Fuzzy trait |
| Ground searching | Percentage of use of ground searching tactic for collecting food items (i.e. species obtaining food from the ground) | Feeding strategy | Fuzzy trait |
| Fruit taking | Percentage of use of fruit taking tactic for collecting food items (i.e. species taking fruits or other plant materials from trees or ground) | Feeding strategy | Fuzzy trait |
| Nectar taking | Percentage of use of nectar taking tactic from flowers | Feeding strategy | Fuzzy trait |

**Table S2.** Information on quality of the functional spaces.

| **Trait category** | **Number of axes** | **Deviation weighting value** |
| --- | --- | --- |
| Morphological traits | 4 | mad = 0.022 |
| Diet | 5 | mad = 0.0022 |
| Foraging stratum | 3 | mad = 0.04 |
| Feeding strategy | 6 | mad = 0.033 |
| Traits combined | 8 | mad = 0.035 |

**Table S3.** Phylogenetic signal in species’ morphological traits. All *K* values (Value) are greater than 1, which indicates that the observed phylogenetic signal is higher than null expectations from Brownian motion.

| **Funtional trait** | **Value** |
| --- | --- |
| Wing length | 1.83 |
| Tail length | 1.94 |
| Culmen | 1.30 |
| Tarsus length | 1.82 |
| Body mass | 1.48 |

**Table S4.** Summary of regression models relating observed functional diversity metrics to elevation, including both segmented and simple linear regressions. For segmented regressions, estimated breakpoints and their corresponding slopes (Slope 1 and Slope 2) with 95% confidence intervals are shown, whereas for simple linear regressions, slope estimates and 95% confidence intervals are listed under Slope 1.

|  | **Estimated breakpoint** | **Confidence Intervals**  **(Slope 1)** | | **Confidence Intervals**  **(Slope 2)** | |  |
| --- | --- | --- | --- | --- | --- | --- |
| **Metric** | **(± SE)** | Lower | Upper | Lower | Upper | **R^2^** |
| Morphological traits | |  |  |  |  |  |
| **FRic** | - | -0.03  (-0.03) | -0.02  (-0.03) | - | - | 0.91 |
| **FNND** | 883.297 m  (± 157.943 m) | -3.464 × 10^-4^  (-1.105 × 10^-4^) | 1.255 × 10^-4^  (-1.105 × 10^-4^) | 8.619 × 10^-5^  (-1.493 × 10^-4^) | 2.123 × 10^-5^  (-1.493 × 10^-4^) | 0.88 |
| **FEve** | 1888.551 m  (± 66.185 m) | 1.713 × 10^-5^  (3.670 × 10^-5^) | 5.629 × 10^-5^  (3.670 × 10^-5^) | -3.827 × 10^-4^  (-2.033 × 10^-4^) | -2.388 × 10^-5^  (-2.033 × 10^-4^) | 0.83 |
| Dietary traits | |  |  |  |  |  |
| **FRic** | 1942.26 m  (± 37.17 m) | -5.395 × 10^-7^  (-2.815 × 10^-7^) | -2.347 × 10^-7^  (-2.815 × 10^-7^) | 2.118 × 10^-6^  (4.483 × 10^-6^) | 6.848 × 10^-6^  (4.483 × 10^-6^) | 0.84 |
| **FNND** | 1912.079 m  (± 62.53 m) | 8.867 × 10^-6^  (1.871 × 10^-5^) | 2.856 × 10^-5^  (1.871 × 10^-5^) | 4.694 × 10^-5^  (1.372 × 10^-4^) | 2.275 × 10^-4^  (1.372 × 10^-4^) | 0.95 |
| **FEve** | - | 9.04 × 10^-5^  (1.27 × 10^-4^) | 0.0002  (1.27× 10^-4^) | - | - | 0.88 |
| Foraging strata | |  |  |  |  |  |
| **FRic** | - | -1 × 10^-5^  (-8 × 10^-6^) | -1.35 × 10^-6^  (-8 × 10^-6^) | - | ^-^ | 0.49 |
| **FNND** | 669.89 m  (± 130.93 m) | -8.121 × 10^-5^  (-2.149 × 10^-5^) | 3.823 × 10^-5^  (-2.149 × 10^-5^) | 2.064 × 10^-5^  (2.716 × 10^-5^) | 3.367 × 10^-5^  (2.716 × 10^-5^) | 0.96 |
| **FEve** | 605.78 m  (± 79.14 m) | -0.0006  (-0.0003) | 0.0001  (-0.0003) | 0.0001  (0.0002) | 0.0002  (0.0002) | 0.96 |
| Feeding strategy | |  |  |  |  |  |
| **FRic** | - | -6.388 × 10^-7^  (-5.03 × 10^-7^) | -3.678 × 10^-7^  (-5.03 × 10^-7^) | - | - | 0.90 |
| **FNND** | 1665.83 m  (± 114.26 m) | 6.705 × 10^-6^  (2.219 × 10^-5^) | 3.767 × 10^-5^  (2.219 × 10^-5^) | 4.595 × 10^-5^  (1.039 × 10^-4^) | 1.618 × 10^-4^  (1.039 × 10^-4^) | 0.95 |
| **FEve** | - | 5.217 × 10^-5^  (8.081 × 10^-5^) | 1.094 × 10^-4^  (8.081 × 10^-5^) | - | - | 0.84 |
| All traits | |  |  |  |  |  |
| **FRic** | - | -5.508 × 10^-12^  (-2.943 × 10^-12^) | -3.771 × 10^-13^  (-2.943 × 10^-12^) | - | - | 0.47 |
| **FNND** | 1283.65 m  (± 161.20 m) | -7.089 × 10^-6^  (1.394 × 10^-6^) | 9.878 × 10^-6^  (1.394 × 10^-6^) | 1.041 × 10^-5^  (1.890 × 10^-5^) | 2.738 × 10^-5^  (1.890 × 10^-5^) | 0.93 |
| **FEve** | - | 2.229 × 10^-5^  (3.985 × 10^-5^) | 5.740 × 10^-5^  (3.985 × 10^-5^) | - | - | 0.77 |

**
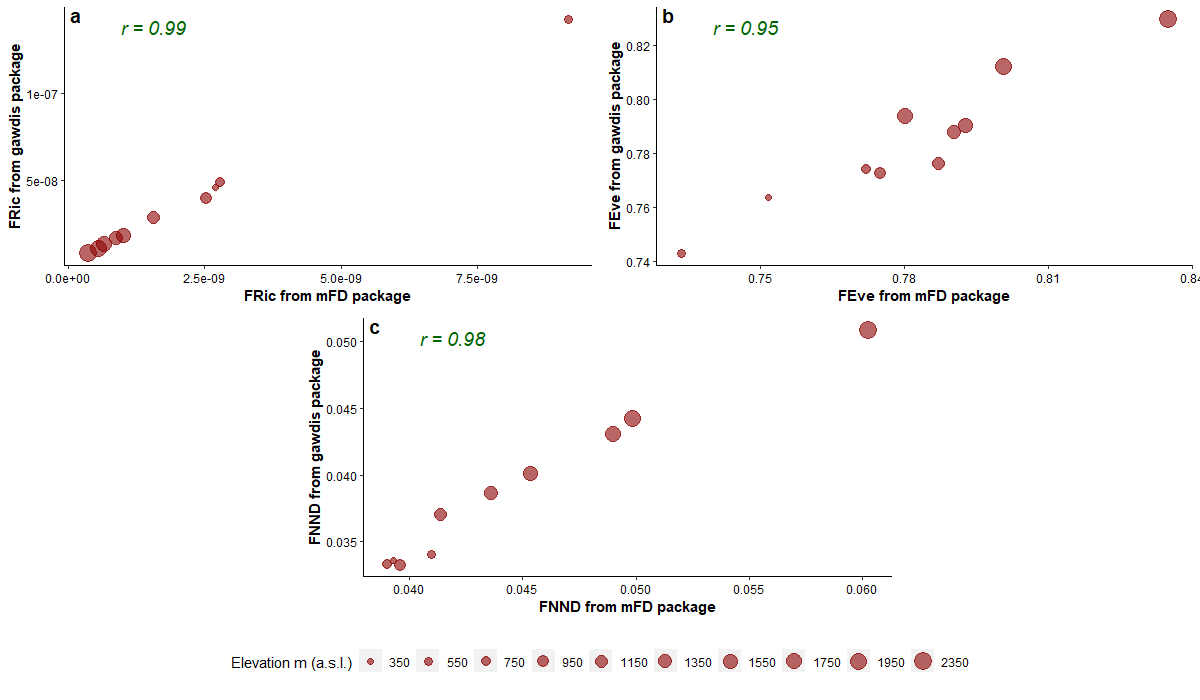
**

**Fig. S2.** A plot of each functional diversity metric based on all types of traits, calculated using the mFD package against the same metric calculated using the *gawdis* package. Note that all metrics computed through the *mFD* package are highly and significantly correlated with those calculated through the *gawdis* package.

**
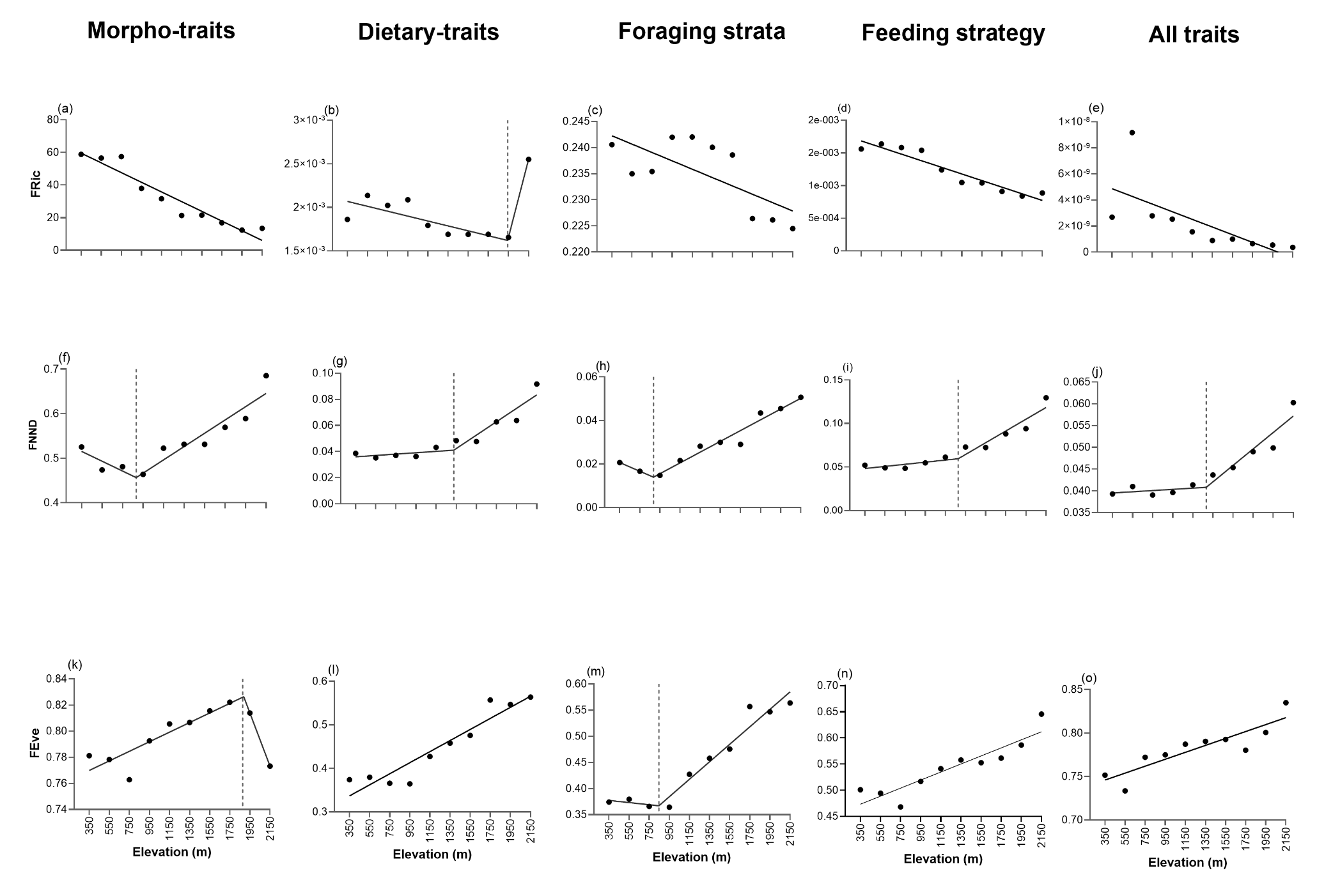
**

**Figure S3.** Elevational gradient in functional diversity, measured as functional richness (a-e), functional nearest neighbour distance (f-j), and functional evenness (k-o). Black points represent observed data, solid black lines indicate either linear or segmented regressions, and dashed vertical lines mark breakpoints where segmented regressions were fitted.

**Table S5.** Summary of regression models relating effect sizes of functional diversity metrics to elevation, including both segmented and simple linear regressions. For segmented regressions, estimated breakpoints and their corresponding slopes (Slope 1 and Slope 2) with 95% confidence intervals are shown, whereas for simple linear regressions, slope estimates and 95% confidence intervals are listed under Slope 1.

|  | **Estimated breakpoint** | **Confidence Intervals**  **(Slope 1)** | | **Confidence Intervals**  **(Slope 2)** | |  |
| --- | --- | --- | --- | --- | --- | --- |
| **Metric** | **(± SE)** | Lower | Upper | Lower | Upper | **R^2^** |
| Morphological traits | |  |  |  |  |  |
| **SES.FRic** | 987.55 m  (± 113.47 m) | -0.008  (-0.005) | -0.002  (-0.005) | -0.0002  (0.001) | 0.003  (0.001) | 0.79 |
| **SES.FNND** | 879.35 m  (± 192.66 m) | -0.008  (-0.004) | 0.001  (-0.004) | -0.0007  (0.0005) | 0.002  (0.0005) | 0.63 |
| **SES.FEve** | - | -0.001  (-9.777 × 10^-5^) | 0.0008  (-9.777 × 10^-5^) | - | - | 0.008 |
| Dietary traits | |  |  |  |  |  |
| **SES.FRic** | 1922.60 m  (± 28.72 m) | 0.0008  (0.001) | 0.0024  (0.001) | 0.011  (0.016) | 0.021  (0.016) | 0.98 |
| **SES.FNND** | - | 0.0003  (0.0008) | 0.001  (0.0008) | - | - | 0.62 |
| **SES.FEve** | - | 0.0002  (0.0008) | 0.001  (0.0008) | - | - | 0.54 |
| Foraging strata | |  |  |  |  |  |
| **SES.FRic** | - | -0.0004  (0.0006) | 0.001  (0.0006) | - | - | 0.18 |
| **SES.FNND** | - | -0.0005  (0.0007) | 0.002  (0.0007) | - | - | 0.18 |
| **SES.FEve** | - | -0.001  (-0.0002) | 0.0008  (-0.0002) | - | - | 0.03 |
| Feeding strategy | |  |  |  |  |  |
| **SES.FRic** | 1497.53 m  (± 84.62 m) | -0.002  (-0.001) | 0.0003  (-0.001) | 0.001  (0.002) | 0.004  (0.002) | 0.86 |
| **SES.FNND** | - | 0.0006  (0.001) | 0.001  (0.001) | - | - | 0.78 |
| **SES.FEve** | - | -0.0007  (0.0001) | 0.0009  (0.0001) | - | - | 0.01 |
| All traits | |  |  |  |  |  |
| **SES.FRic** | - | -0.0009  (-1.423) | 0.0009  (-1.423) | - | - | 1.5 × 10^-6^ |
| **SES.FNND** | - | -0.0006  (4.206 × 10^-5^) | 0.0007  (4.206 × 10^-5^) | - | - | 0.002 |
| **SES.FEve** | - | 3.94 × 10^-5^  (0.001) | 0.002  (0.001) | - | - | 0.42 |

**Table S6.** Summary of regression models relating observed phylogenetic diversity metrics and their effect sizes (SES) to elevation, including both segmented and simple linear regressions. For segmented regressions, estimated breakpoints and their corresponding slopes (Slope 1 and Slope 2) with 95% confidence intervals are shown, whereas for the simple linear regression, slope estimate and 95% confidence intervals are listed under Slope 1.

|  | **Estimated breakpoint** | **Confidence Intervals**  **(Slope 1)** | | **Confidence Intervals**  **(Slope 2)** | |  |
| --- | --- | --- | --- | --- | --- | --- |
| **Metric** | **(± SE)** | Lower | Upper | Lower | Upper | **R^2^** |
|  | |  |  |  |  |  |
| **MPD** | - | -0.004  (-0.001) | 1.841 × 10^-3^  (-0.001) | - | - | 0.09 |
| **MNTD** | 1943.06 m  (± 53.36 m) | 0.001  (0.0043) | 0.008  (0.0043) | 0.017  (0.0495) | 0.082  (0.0495) | 0.91 |
| **SES.MPD** | - | -0.001  (-0.0001) | 0.0008  (-0.0001) | - | - | 0.02 |
| **SES.MNTD** | - | -0.0005  (0.0004) | 0.001  (0.0004) | - | - | 0.11 |

**
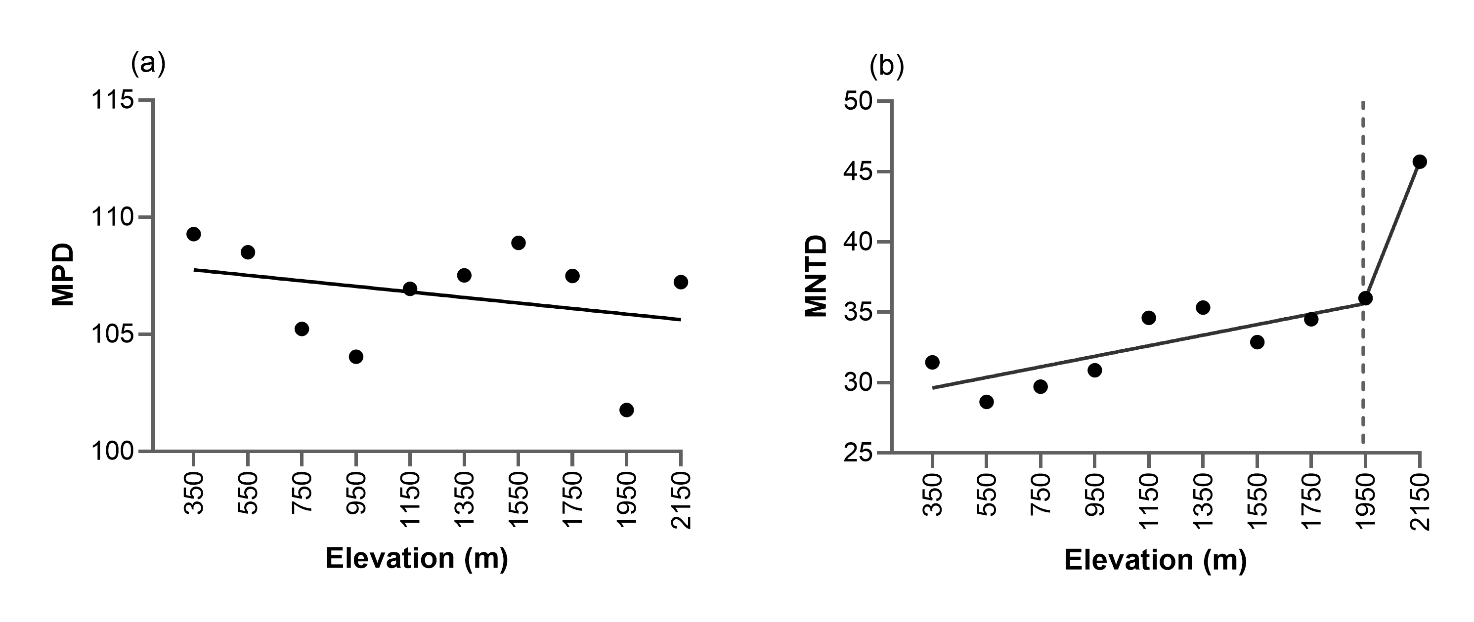
**

**Figure S4.** Elevational gradient in observed phylogenetic diversity measured as mean pairwise distance (a) and mean nearest taxonomic distance (b). Black points represent observed data, solid black lines indicate either linear or segmented regressions and the dashed vertical line marks breakpoint where segmented regression was fitted.

**Appendix S3**

Based on Pigot et al. (2016), we employed a greedy-search algorithm to investigate how the disposition of species in trait space represented by the pool of all functional traits changes when transitioning from elevations with lower species richness to those with higher richness. The approach involved systematically eliminating species from the richer-species assemblage that were not present in the poorer-species assemblage, which when removed resulted in the greatest reduction in the convex hull volume until the volume reached or fell below that of the poorer-species assemblage. The count of species eliminated during this process represented the contribution of niche expansion to the increase in species richness, while any remaining species in the richer-species assemblage contributed to the overall niche packing pattern (Fig. S5).


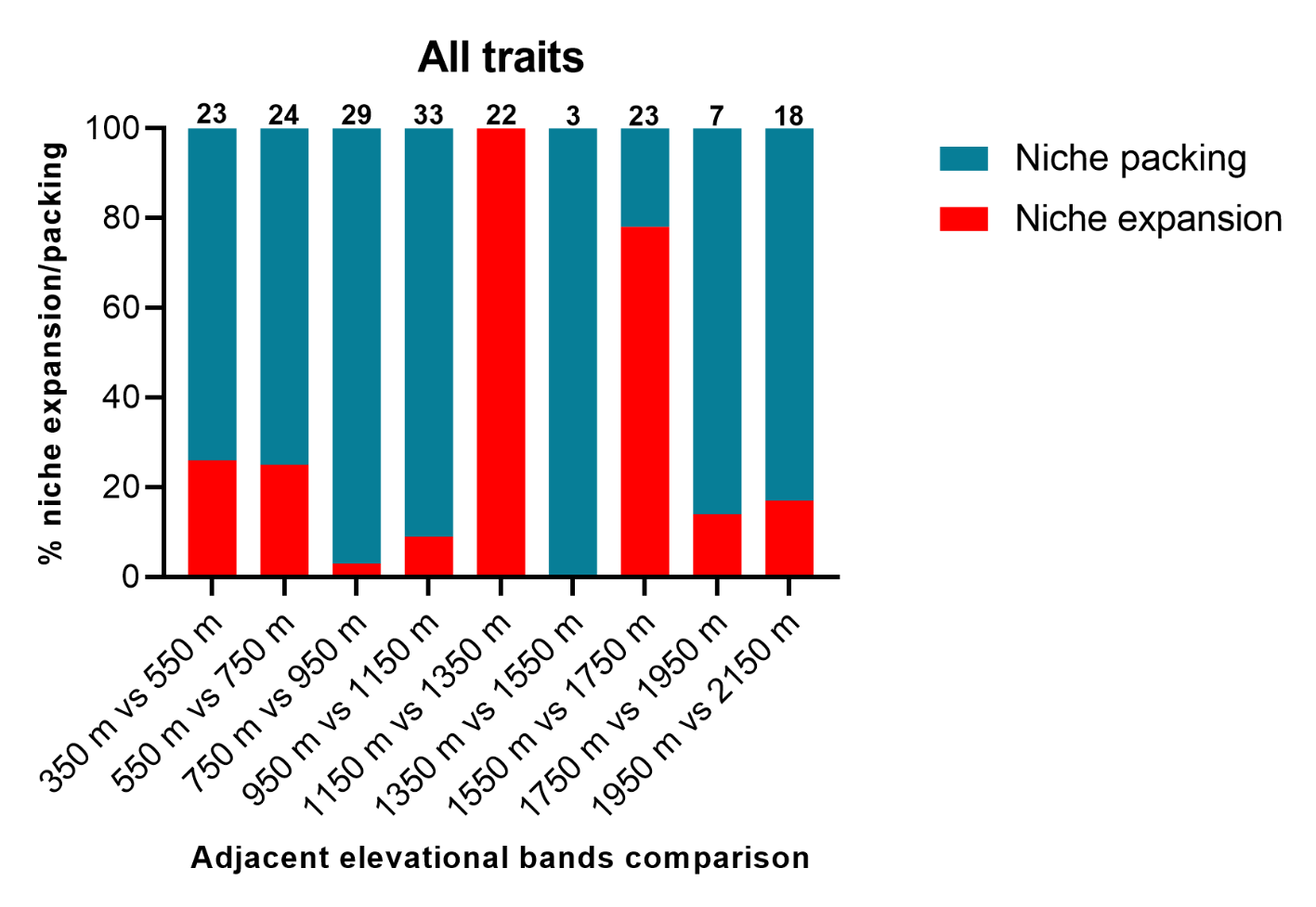


**Figure S5.** Trait-space packing/expansion over the elevational gradient of Mt. Cameroon. Each value is computed as the proportion of unique species contributing to packing/expansion for two adjacent assemblages, that is the proportion of unique species needed to accommodate the convex hull volume of the richer-species assemblage within the species-poorer assemblage. The number of unique species contributing to packing/expansion for two adjacent assemblages is shown above the bars. Convex hull volumes are estimated by pooling all functional trait categories.

**References**

Blomberg, S. P., Garland Jr, T., & Ives, A. R. (2003). Testing for phylogenetic signal in comparative data: Behavioral traits are more labile. *Evolution*, *57*(4), 717–745.

Cornwell, W. K., Schwilk, D. W., & Ackerly, D. D. (2006). A trait‐based test for habitat filtering: Convex hull volume. *Ecology*, *87*(6), 1465–1471.

Kembel, S. W., Cowan, P. D., Helmus, M. R., Cornwell, W. K., Morlon, H., Ackerly, D. D., Blomberg, S. P., & Webb, C. O. (2010). Picante: R tools for integrating phylogenies and ecology. *Bioinformatics*, *26*(11), 1463–1464.

Magneville, C., Loiseau, N., Albouy, C., Casajus, N., Claverie, T., Escalas, A., Leprieur, F., Maire, E., Mouillot, D., & Villéger, S. (2022). mFD: An R package to compute and illustrate the multiple facets of functional diversity. *Ecography*, *2022*(1). https://doi.org/10.1111/ecog.05904

Maire, E., Grenouillet, G., Brosse, S., & Villéger, S. (2015). How many dimensions are needed to accurately assess functional diversity? A pragmatic approach for assessing the quality of functional spaces. *Global Ecology and Biogeography*, *24*(6), 728–740. https://doi.org/10.1111/geb.12299

Montaño-Centellas, F. A., Loiselle, B. A., & Tingley, M. W. (2021). Ecological drivers of avian community assembly along a tropical elevation gradient. *Ecography*, *44*(4), 574–588. https://doi.org/10.1111/ecog.05379

Muggeo, V. M., & Muggeo, M. V. M. (2017). Package ‘segmented.’ *Biometrika*, *58*(525–534), 516.

Pigot, A. L., Trisos, C. H., & Tobias, J. A. (2016). Functional traits reveal the expansion and packing of ecological niche space underlying an elevational diversity gradient in passerine birds. *Proceedings of the Royal Society B: Biological Sciences*, *283*(1822), 20152013.

Sedláček, O., Pernice, R., Ferenc, M., Mudrová, K., Motombi, F. N., Albrecht, T., & Hořák, D. (2023). Abundance variations within feeding guilds reveal ecological mechanisms behind avian species richness pattern along the elevational gradient of Mount Cameroon. *Biotropica*, *55*(3), 706–718.

Villéger, S., Mason, N. W. H., & Mouillot, D. (2008a). New multidimensional functional diversity indices for a multifaceted framework in functional ecology. *Ecology*, *89*(8), 2290–2301.

Villéger, S., Mason, N. W., & Mouillot, D. (2008b). New multidimensional functional diversity indices for a multifaceted framework in functional ecology. *Ecology*, *89*(8), 2290–2301.

Webb, C. O., Ackerly, D. D., McPeek, M. A., & Donoghue, M. J. (2002). Phylogenies and community ecology. *Annual Review of Ecology and Systematics*, *33*(1), 475–505.

Weiher, E., Clarke, G. P., & Keddy, P. A. (1998). Community assembly rules, morphological dispersion, and the coexistence of plant species. *Oikos*, 309–322.
